# Prenatal Polybrominated Diphenyl Ether Exposure and Body Mass Index in Children Up To 8 Years of Age

**DOI:** 10.1289/EHP139

**Published:** 2016-06-10

**Authors:** Ann M. Vuong, Joseph M. Braun, Andreas Sjödin, Glenys M. Webster, Kimberly Yolton, Bruce P. Lanphear, Aimin Chen

**Affiliations:** 1 Division of Epidemiology, Department of Environmental Health, University of Cincinnati College of Medicine, Cincinnati, Ohio, USA; 2 Department of Epidemiology, Brown University School of Public Health, Providence, Rhode Island, USA; 3 Division of Laboratory Sciences, National Center for Environmental Health, Centers for Disease Control and Prevention, Atlanta, Georgia, USA; 4 BC Children’s Hospital Research Institute, Simon Fraser University, Vancouver, British Columbia, Canada; 5 Division of General and Community Pediatrics, Department of Pediatrics, Cincinnati Children’s Hospital Medical Center, Cincinnati, Ohio, USA

## Abstract

**Background:**

Prenatal exposure to endocrine disruptors has been associated with increased risk of childhood obesity. However, epidemiologic studies on polybrominated diphenyl ethers (PBDEs) are limited despite animal studies indicating PBDEs’ potential role as an obesogen.

**Objectives:**

We investigated whether maternal concentrations of BDEs 28, 47, 99, 100, 153, and ΣPBDEs during pregnancy were associated with anthropometric measures in children aged 1–8 years.

**Methods:**

We examined 318 mother–child pairs in the Health Outcomes and Measures of the Environment (HOME) Study, a birth cohort enrolled from 2003 through 2006 (Cincinnati, OH). Serum PBDEs were measured at 16 ± 3 weeks gestation. We measured child height (1–8 years), weight (1–8 years), body mass index (BMI) (2–8 years), waist circumference (4–8 years), and body fat (8 years). To account for repeated measures, we used linear mixed models and generalized estimating equations to estimate associations between maternal PBDEs and child anthropometric measures.

**Results:**

We found no statistically significant associations between prenatal PBDEs and height or weight z-score. A 10-fold increase in maternal serum BDE-153 was associated with lower BMI z-score (β = –0.36; 95% CI: –0.60, –0.13) at 2–8 years, smaller waist circumference (β = –1.81 cm; 95% CI: –3.13, –0.50) at 4–8 years, and lower percent body fat (β = –2.37%; 95% CI: –4.21, –0.53) at 8 years. A decrease in waist circumference at 4–8 years was observed with a 10-fold increase in BDE-100 (β = –1.50 cm; 95% CI: –2.93, –0.08) and ΣPBDEs (β = –1.57 cm; 95% CI: –3.11, –0.02).

**Conclusions:**

Reverse causality may have resulted in prenatal PBDEs, particularly BDE-153, and decreased BMI, waist circumference, and body fat.

**Citation:**

Vuong AM, Braun JM, Sjödin A, Webster GM, Yolton K, Lanphear BP, Chen A. 2016. Prenatal polybrominated diphenyl ether exposure and body mass index in children up to 8 years of age. Environ Health Perspect 124:1891–1897; http://dx.doi.org/10.1289/EHP139

## Introduction

Childhood obesity is associated with adverse health effects, including diabetes, dyslipidemia, fatty liver disease, and hypertension (Daniels 2009). Between 1980 and 2013, the global prevalence of childhood obesity increased by about 50% (Ng et al. 2014). Obesity is attributable to a convergence of several factors, such as genetic predisposition, excess food quantity, readily available energy-dense food, and less energy expenditure, but recent evidence implicates a number of endocrine-disrupting chemicals as potential obesogens, including bisphenol A (BPA), dichlorodiphenyldichloroethylene (DDE), polychlorinated biphenyls (PCBs), and phthalates (Braun et al. 2014; Valvi et al. 2013; Verhulst et al. 2009; Warner et al. 2014).

Polybrominated diphenyl ethers (PBDEs) are a class of endocrine-disrupting flame retardants used in a number of household and industrial products, including electronics, polyurethane foams, and textiles. Their ability to persist, bioaccumulate, and biomagnify has resulted in chronic and prolonged exposure despite the voluntary cessation of penta- and octaBDE manufacturing in the United States in 2004. PBDEs have been associated with weight gain in animal studies (Bondy et al. 2013; Dufault et al. 2005; Fernie et al. 2006; Gee and Moser 2008; Suvorov et al. 2009). PBDEs have been reported to increase adipocyte differentiation, decrease glucose oxidation, disturb glucose homeostasis, and alter gene expression in the metabolic pathways by directly interacting with retinoic X receptor (RXR), a key regulatory transcription factor in the adipogenic pathway in vertebrates (Bastos Sales et al. 2013; Hoppe and Carey 2007; Kamstra et al. 2014; Suvorov and Takser 2010; Tung et al. 2014). In addition, one study reported that BDE-47 activates peroxisome proliferator-activated receptor gamma (PPAR-γ) in 3T3-L1 cells, a key regulator of adipogenesis in vertebrates (Auwerx 1999; Kamstra et al. 2014).

Previous epidemiologic studies examining PBDEs by body mass index (BMI) status have reported conflicting findings (Leijs 2010; Lim et al. 2008; Turyk et al. 2010; Windham et al. 2010). These studies focused primarily on postnatal rather than prenatal exposures to PBDEs. Insults to the developing fetus by endocrine disruptors may influence offspring growth. Plasma insulin-like growth factor 1 (IGF-1) was elevated in male rats perinatally exposed to low doses of BDE-47 (Suvorov et al. 2009). IGF-1 gene expression alteration has been shown to play a role in glucose metabolism and gestational programming of obesity (Ross et al. 2007). Further, epidemiologic studies have reported that prenatal PBDE concentrations disrupt thyroid hormone homeostasis, which is involved in growth and development, accelerating basal energy expenditure, lipid metabolism, and thermogenesis (Abdelouahab et al. 2013; Lim et al. 2008; Pucci et al. 2000; Stapleton et al. 2011; Vuong et al. 2015). Only one study has examined PBDE exposure *in utero* and child measures of growth in a cohort of Mexican-American children and reported sex-specific associations (Erkin-Cakmak et al. 2015). In the present study, we examined the relation between maternal PBDE concentrations at approximately 16 weeks of gestation and child anthropometric measures at ages 1–8 years, including height, weight, BMI, waist circumference, and body fat percentage.

## Methods

### Study Participants and Design

The Health Outcomes and Measures of the Environment (HOME) Study is an ongoing prospective birth cohort that enrolled women between March 2003 and February 2006 from nine prenatal clinics located in the greater Cincinnati, Ohio, area (USA). Eligibility criteria included being ≥ 18 years of age, living in a house constructed before 1978 (a criterion relating to a goal of the randomized trial examining lead and injury hazard reduction interventions), intending to continue prenatal care and deliver at one of the nine collaborating obstetric practices and hospitals, being HIV negative, and not receiving seizure, thyroid, or chemotherapy/radiation medications. A total of 390 women of the 468 enrolled remained to deliver live singleton infants. Our study focused on 318 mother–child pairs that had concentrations of PBDEs measured at approximately 16 weeks of gestation and at least one measure of child anthropometry at age 1, 2, 3, 4, 5, or 8 years. The study protocol was approved by the institutional review boards at the Cincinnati Children’s Hospital Medical Center and the Centers for Disease Control and Prevention (CDC).

### PBDE Assessment

Maternal serum samples were collected at 16 ± 3 weeks of gestation and stored at –80°C. Concentrations of BDEs 17, 28, 47, 66, 85, 99, 100, 153, 154, and 183 were measured using gas chromatography/isotope dilution high-resolution mass spectrometry at the CDC (Jones et al. 2012; Sjödin et al. 2004). Serum samples were pretreated and extracted by solid phase extraction. Each batch of serum samples included three quality control and three method blank samples. The limit of detection (LOD) was defined as three times the standard deviation (SD) of the method blanks analyzed in parallel with the study samples or as 0.5 pg/μL (in 10 μL nonane) (in the absence of detectable blanks). Total serum lipids were based on measurements of triglycerides and total cholesterol using standard enzymatic methods (Phillips et al. 1989). Serum PBDE values < LOD were substituted with the LOD divided by the square root of 2 (Hornung and Reed 1990). We focused our analysis on congeners with detection frequencies > 80% (BDEs 28, 47, 99, 100, and 153) and the sum of these congeners (ΣPBDEs).

### Child Anthropometry

Child height, weight, and waist circumference were measured in triplicate each visit; we used an average of the three measurements. Height and weight were obtained at 1, 2, 3, 4, 5, and 8 years of age. The Ayrton Stadiometer Model S100 was used to measure height (to the nearest 0.1 cm), with the child standing straight without shoes or a head covering on, positioned with the heels against the wall. If children were not standing independently at the 1-year visit, recumbent length was measured with a standard infant length board. For weight (to the nearest 0.01 kg), children were either in undergarments or a dry diaper on the infant ScaleTronix scale (White Plains, NY) or the ScaleTronix Pediatric Scale Model 4802. Age- and sex-specific height and weight *z*-scores (1, 2, 3, 4, 5, and 8 years) as well as BMI *z*-scores (2, 3, 4, 5, 8 years) were calculated based on U.S. references from the National Center for Health Statistics (Kuczmarski et al. 2000). BMI *z*-scores ≥ 85th percentile were considered overweight or obese. At ages 4, 5, and 8 years, waist circumference (cm) was measured by placing a plastic measuring tape around the abdomen at the level of the iliac crest. Body fat percentage was measured at 8 years of age via bioelectrical impedance analysis using the Tanita children’s body fat monitor (Arlington Heights, IL).

### Statistical Analyses

PBDEs were log_10_-transformed to reduce the influence of extreme values. Linear mixed models with an unstructured correlation matrix and a random intercept were used to estimate β coefficients and 95% confidence intervals (CIs) for individual BDE congeners and ΣPBDEs in relation to child height and weight *z*-scores at 1–8 years of age, BMI *z*-scores at 2–8 years of age, and waist circumference at 4–8 years of age. Interaction terms between PBDEs (continuous) and child age (categorical) were included in the models to determine whether child growth differed over time. However, because interaction terms were not statistically significant (*p* > 0.10), overall estimates are provided for height and weight *z*-scores at 1–8 years; BMI *z*-scores at 2–8 years; and waist circumference at 4–8 years. The relation between PBDEs and having a high-end BMI *z*-score (≥ 85th percentile) or a low-end BMI *z*-score (≤ 15th percentile) at ages 2–8 years was examined using generalized linear models (GLM) with generalized estimating equations (GEE) to estimate odds ratios (ORs) and 95% CIs. Multiple linear regression models were used to examine the association between individual BDE congeners and ΣPBDEs and body fat percentage at 8 years. Dose response was examined using generalized additive models to examine linearity for PBDEs and child growth measures. Because the results did not indicate a nonmonotonic relationship, the associations between tertiles of PBDE concentrations and child anthropometric measures were assessed for linear trend using the median value in each tertile as a continuous variable in the previously described models (Greenland 1995). We also examined whether effect modification by child sex was present by including the interaction term of PBDEs (continuous) and child sex in the models, with *p* < 0.10 considered significant. As a sensitivity analysis, we adjusted by other environmental contaminants in separate models, including maternal serum concentrations of DDE, lead (Pb), perfluorooctanoate (PFOA), perfluorooctane sulfonate (PFOS), and ΣPCBs (sum of PCB congeners 28, 74, 99, 105, 118, 146, 153, 156, 170, 180, 183, 187, 194, 199, and 206) and creatinine adjusted maternal urinary concentrations of BPA and di(2-ethylhexyl) phthalate (DEHP).

Covariates included in the final regression models were based on results of bivariate analyses examining the relationship with child growth (*p* < 0.20). Final models included the following covariates (categorized as shown in Table 1): maternal age at enrollment, race, education, family income, maternal depression (assessed by Beck Depression Inventory II at ~ 16 weeks of gestation) (Beck et al. 1996), maternal serum cotinine (ng/mL, continuous) at enrollment, and fresh fruit and vegetable intake during pregnancy. Additional covariates were included in models examining height *z*-scores (maternal height); weight *z*-scores (maternal prepregnancy weight); BMI *z*-scores (prepregnancy BMI); and waist circumference and body fat percentage (prepregnancy BMI, child sex, and child age). Two of the mother–child pairs were missing information on maternal depression and thus were not included in the final analyses. As a secondary analysis, we examined whether prenatal PBDEs impacted growth trajectories by regressing on the differences in *z*-scores of child growth measures. We additionally estimated β coefficients without adjustment for prepregnancy maternal anthropometric measures to determine whether our findings differed if we did not take into account maternal measures of height, weight, and BMI. As an additional sensitivity analysis, we adjusted for potential selection bias by applying weights to the regression models equal to the inverse probability of being observed. Stata version 12.1 (StataCorp, College Station, TX) was used for statistical analyses, and graphs were produced using GraphPad Prism (GraphPad, San Diego, CA).

**Table 1:** Maternal serum concentrations of ∑PBDEs (ng/g lipid) and child anthropometric measures at 8 years by maternal and child characteristics, HOME Study.

Characteristic	∑PBDEs		BMI *z*-score		Waist circumference		Body fat percentage	
	*n*	GM (GSD)	*n*	Mean ± SD	*n*	Mean ± SD	*n*	Mean ± SD
Maternal age (years)
< 25	64	47.9 (2.2)*	55	0.37 ± 0.95	54	59.2 ± 7.1	54	21.1 ± 6.5
25–34	175	39.2 (2.7)*	129	0.37 ± 0.98	127	61.0 ± 7.6	127	20.7 ± 5.9
≥ 35	48	29.6 (2.5)*	32	0.26 ± 1.03	30	62.0 ± 11.7	29	20.8 ± 6.8
Race/ethnicity
Non-Hispanic white	188	33.4 (2.5)*	131	0.19 ± 0.94*	127	60.6 ± 7.5	127	19.7 ± 5.5*
Non-Hispanic black and others	99	52.8 (2.5)*	85	0.60 ± 0.98*	84	60.8 ± 9.1	83	22.5 ± 6.8*
Education
High school or less	70	53.0 (2.2)*	57	0.69 ± 0.89*	56	61.0 ± 8.3	56	23.1 ± 6.5*
Some college/2-year degree	73	42.1 (2.3)*	56	0.06 ± 1.07*	56	58.8 ± 7.5	55	19.0 ± 5.6*
Bachelor’s	90	34.0 (2.6)*	65	0.39 ± 0.95*	63	62.1 ± 9.1	63	20.9 ± 6.0*
Graduate or professional	54	30.2 (3.0)*	38	0.22 ± 0.87*	36	60.5 ± 6.7	36	19.7 ± 5.8*
Family income
< $40,000	108	51.7 (2.5)*	88	0.50 ± 1.06	88	60.5 ± 9.0	87	21.8 ± 7.0
$40,000–$79,999	99	36.6 (2.5)*	72	0.24 ± 0.92	69	60.8 ± 8.1	69	20.1 ± 5.7
≥ $80,000	80	29.1 (2.4)*	56	0.27 ± 0.90	54	60.7 ± 6.7	54	20.1 ± 5.1
Maternal depression
Minimal/mild	261	37.5 (2.5)*	196	0.32 ± 0.96	192	60.4 ± 8.2	191	20.5 ± 6.0*
Moderate/severe	24	64.1 (2.9)*	19	0.72 ± 1.09	18	63.5 ± 7.2	18	24.4 ± 6.9*
Maternal smoking
None	245	37.2 (2.6)	181	0.29 ± 0.95	177	60.3 ± 7.6	177	20.4 ± 5.8
Environmental tobacco smoke	20	47.8 (2.2)	18	0.56 ± 1.20	18	61.8 ± 9.2	18	23.4 ± 8.1
Active	22	56.5 (2.6)	17	0.81 ± 0.89	16	63.7 ± 12.2	15	22.2 ± 6.6
Prepregnancy BMI
Underweight/normal	147	35.4 (2.5)	111	0.13 ± 0.95*	106	59.3 ± 7.1*	106	19.7 ± 5.6*
Overweight	79	39.6 (2.6)	56	0.33 ± 0.92*	56	60.0 ± 6.7*	55	19.9 ± 4.5*
Obese	61	48.9 (2.7)	49	0.89 ± 0.92*	49	64.2 ± 10.6*	49	24.1 ± 7.6*
Marital status
Married/living with partner	225	35.6 (2.6)*	160	0.24 ± 0.94*	155	60.3 ± 7.7	155	20.1 ± 5.7*
Not married, living alone	62	55.3 (2.1)*	56	0.67 ± 1.04*	56	61.6 ± 9.3	55	22.7 ± 7.1*
Fresh fruit and vegetable intake during pregnancy
≥ Daily	233	38.7 (2.6)	170	0.37 ± 0.98	166	61.1 ± 8.1	165	21.1 ± 6.3
< Daily	54	41.1 (2.3)	46	0.29 ± 0.98	45	59.1 ± 8.4	45	19.7 ± 5.6
Child sex
Male	129	36.8 (2.6)	95	0.24 ± 0.95	93	59.0 ± 7.0*	93	18.4 ± 4.4*
Female	158	41.2 (2.6)	121	0.44 ± 0.99	118	62.0 ± 8.7*	117	22.7 ± 6.7*
Note: GM, geometric mean; GSD, geometric standard deviation; SD, standard deviation. Frequencies may not add to the total number of participants because of missing values. *p < 0.05 (two-sided *p-*values using ANOVA or *t*-test).								

## Results

HOME Study mothers were predominantly non-Hispanic white, educated, nonsmokers, married or living with a partner, and had an annual income > $40,000 (Table 1). The most abundant congener was BDE-47, with a geometric mean (GM) of 20.3 ng/g lipid. BDE congeners were highly correlated with each other (*r*_s_ = 0.46–0.92, *p* < 0.0001) (see Tables S1 and S2). Concentrations of ΣPBDEs were lower among women who were older, non-Hispanic white, highly educated, of a higher income, minimally/mildly depressed, and married/living with a partner. BMI *z*-scores were significantly higher among children whose mothers were non-Hispanic black and others, less educated, obese before conception, and not married/living alone. At age 8 years, both waist circumference and body fat percentage were higher among female children and those with mothers who were obese before pregnancy. At age 8 years, approximately 25% of HOME Study children were either overweight or obese (see Table S3). Average (± SD) waist circumference and body fat percentage were 60.7 ± 8.2 cm and 20.8 ± 6.1%, respectively. Participants included in the analysis were significantly more likely to be older, non-Hispanic white, educated, of a higher income, and nonsmokers compared with women excluded due to insufficient information on PBDEs (*n* = 30) and/or child anthropometric measures (*n* = 42) (see Table S4).

Maternal concentrations of PBDEs were not associated with weight or height *z*-scores in children ages 1–8 years (Table 2). Although we observed a decrease in BMI *z*-scores in children ages 2–8 years with a 10-fold increase across all BDE congeners and ΣPBDEs, only BDE-153 was statistically significant (β = –0.36; 95% CI: –0.60, –0.13). An inverse association was also observed between BDE-100 (β = –1.50 cm; 95% CI: –2.93, –0.08), BDE-153 (β = –1.81 cm; 95% –3.13, –0.50), and ΣPBDEs (β = –1.57 cm; 95% CI: –3.11, –0.02) and waist circumference. Among children 8 years of age, a 10-fold increase in maternal BDE-153 concentration was associated with a 2.37% decrease (95% CI: –4.21, –0.53) in body fat percentage.

**Table 2:** Estimated differences (95% CIs) in child anthropometric measures*^a^* by 10-fold increases in maternal serum concentrations of polybrominated diphenyl ethers (ng/g lipid), HOME Study.*^b^*

PBDEs	Height *z*-scores^*c*^ [β (95% CI)]	Weight *z*-scores^*d*^ [β (95% CI)]	BMI *z*-scores^*e*^ [β (95% CI)]	Waist circumference (cm)^*e*^^,^^*f*^ [β (95% CI)]	Body fat percentage^*e*^^,^^*f*^ [β (95% CI)]
BDE-28	0.03 (–0.26, 0.33)	–0.28 (–0.60, 0.04)	–0.21 (–0.51, 0.10)	–1.09 (–2.77, 0.59)	0.03 (–2.39, 2.45)
BDE-47	0.04 (–0.22, 0.30)	–0.18 (–0.46, 0.10)	–0.08 (–0.35, 0.18)	–0.96 (–2.44, 0.52)	–0.53 (–2.69, 1.64)
BDE-99	0.03 (–0.22, 0.28)	–0.25 (–0.52, 0.02)	–0.18 (–0.45, 0.08)	–1.41 (–2.89, 0.06)	–1.17 (–3.35, 1.02)
BDE-100	–0.001 (–0.25, 0.25)	–0.21 (–0.48, 0.06)	–0.21 (–0.46, 0.05)	–1.50 (–2.93, –0.08)	–1.13 (–3.16, 0.90)
BDE-153	–0.09 (–0.33, 0.14)	–0.24 (–0.49, 0.02)	–0.36 (–0.60, –0.13)	–1.81 (–3.13, –0.50)	–2.37 (–4.21, –0.53)
∑PBDEs	–0.01 (–0.28, 0.26)	–0.28 (–0.57, 0.02)	–0.26 (–0.54, 0.02)	–1.57 (–3.11, –0.02)	–1.50 (–3.75, 0.76)
PBDEs were log_10_-transformed. ^***a***^At 1–8 years of age for weight and length/height *z*-scores, 2–8 years of age for BMI *z*-scores, 4–8 years of age for waist circumference, and 8 years of age for body fat percentage. ^***b***^Adjusted by maternal age, race, education, income, maternal smoking status, maternal depression, fresh fruit and vegetable intake during pregnancy. ^***c***^Additionally adjusted by maternal height. ^***d***^Prepregnancy weight. ^***e***^Prepregnancy BMI. ^***f***^Child sex and child age.					

**Figure 1:**
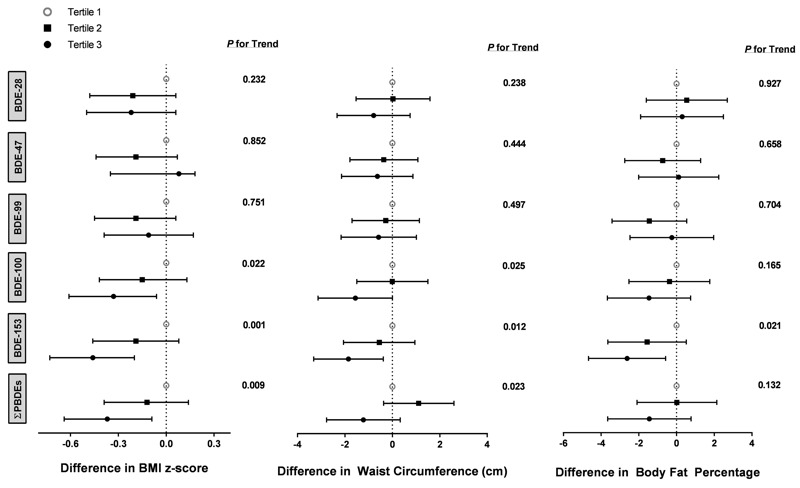
Estimated differences and 95% CIs from multiple linear mixed models for associations between polybrominated diphenyl ether (ng/g lipid) tertiles and BMI *z*-score, waist circumference, or body fat percentage in children. All models were adjusted for maternal age, race, education, income, maternal smoking status, maternal depression, prepregnancy BMI, and maternal fruit and vegetable intake during pregnancy. Waist circumference and body fat percentage models were additionally adjusted for child sex and age (months). Children included in the models were 2–8 years for BMI *z*-score, 4–8 years for waist circumference, and 8 years for body fat percentage. PBDE tertile ranges in ng/g lipid: BDE-28 (0.2–0.7, 0.8–1.3, 1.4–31.4), BDE-47 (1.5–13.1, 13.2–27.9, 28.0–1,290), BDE-99 (0.6–2.9, 3.0–6.0, 6.1–465), BDE-100 (0.4–2.4, 2.5–5.6, 5.7–172), BDE-153 (0.5–3.0, 3.1–6.8, 6.9–152), and ∑PBDEs (4.5–24.9, 25.0–52.9, 53.0–2,047).

There was no significant linear trend between tertiles of PBDEs and weight or height *z*-scores (see Figure S1). However, tertiles of BDE-100, BDE-153, and ΣPBDEs were associated with decreasing BMI *z*-score and waist circumference (Figure 1). The highest tertiles of BDE-100 (β = –0.33; 95% CI: –0.61, –0.06), BDE-153 (β = –0.46; 95% CI: –0.73, –0.20), and ΣPBDEs (β = –0.37; 95% CI: –0.64, –0.09) were significantly associated with decrements in BMI *z*-score. The associations between tertiles of BDE-153 and body fat percentage also presented a decreasing linear trend (*p*_trend_ = 0.021). Although the highest tertile of BDE-100, BDE-153, and ΣPBDEs had the lowest waist circumference and body fat percentage, only the results of BDE-153 tertile was statistically significant. Maternal serum concentrations of BDE-153 ≥ 6.8 ng/g lipid were associated with decreased waist circumference (β = –1.85 cm; 95% CI: –3.32, –0.38) and body fat percentage (β = –2.62%; 95% CI: –4.67, –0.58).

No association was observed between maternal concentrations of PBDEs and being overweight or obese at 2–8 years of age (see Table S5). A 10-fold increase in BDE-153 was associated with higher odds of having a BMI ≤ 15th percentile (OR = 2.18; 95% CI: 1.16, 4.08) (see Table S6). Effect modification by child sex was observed only for the association between maternal BDE-153 concentrations and height *z*-scores (*p*_modification_ = 0.065), with a borderline significant decrease in males (β = –0.32; 95% CI: –0.65, 0.01) but not in females (β = 0.12; 95% CI: –0.20, 0.44) (see Table S7).

No statistically significant associations were observed between prenatal PBDEs and trajectories of child growth (results not shown). Additionally adjusting by BPA, DDE, DEHP, Pb, PFOA, PFOS, or ΣPCBs did not change our overall conclusions (see Table S8). However, significant inverse associations were observed between BDE-28, BDE-153, and ΣPBDEs and weight *z*-score in models adjusting for maternal concentrations of BPA, DDE, or DEHP. A 10-fold increase in BDE-99 was also significantly associated with decreased waist circumference after adjustment for DDE, DEHP, or PFOA. Our conclusions did not change when we did not adjust for maternal anthropometric measures, nor did the results differ with additional adjustment for potential selection bias (results not shown).

## Discussion

We examined the relation between maternal serum concentrations of PBDEs and anthropometric measures in a cohort of children 1–8 years of age residing in Cincinnati, Ohio. We did not observe an increase in child anthropometric measures, including weight *z*-score, height *z*-score, BMI *z*-score, waist circumference, and body fat percentage, with maternal serum concentrations of BDE congeners or ΣPBDEs. In contrast, 10-fold increases in BDE-100, BDE-153, and ΣPBDEs were associated with decreased waist circumference among children 4–8 years old. BDE-153 was also significantly associated with a 0.36 reduction in BMI *z*-score in children 2–8 years of age and a 2.37% reduction in body fat percentage in children 8 years of age.

Previous epidemiologic studies have focused primarily on postnatal exposures of PBDEs (Leijs 2010; Lim et al. 2008; Turyk et al. 2010; Windham et al. 2010). Only one study, the CHAMACOS (Center for the Health Assessment of Mothers and Children of Salinas) Study, has examined pre- and postnatal concentrations of PBDEs and their relation to childhood obesity (Erkin-Cakmak et al. 2015). In the CHAMACOS Study, a significant inverse association was reported between child serum concentrations of PBDEs and BMI and waist circumference *z*-score at 7 years. Specifically, significant decreases in BMI (β = –1.15; 95% CI: –1.53, –0.77) and waist circumference *z*-scores (β = –0.95; 95% CI: –1.26, –0.64) were observed with a 10-fold increase in childhood concentrations of BDE-153. However, Erkin-Cakmak et al. (2015) reported no associations between maternal concentrations of PBDEs and BMI *z*-score, waist circumference *z*-score, or being overweight or obese at 7 years of age. In contrast, we observed a reduction in BMI *z*-score, waist circumference, and body fat percentage with maternal concentrations of BDE-153. Null findings between maternal concentrations of PBDEs and child anthropometric measures reported by Erkin-Cakmak et al. (2015) may be attributable to lower BDE-153 concentrations in the CHAMACOS cohort (GM = 2.4 ng/g lipid; 95% CI: 2.15, 2.69) compared with our study (GM = 5.3 ± 2.9 ng/g lipid). In addition, the prevalence of overweight and obese children in the HOME Study at age 8 years (25%) was lower than that of the CHAMACOS Study at 7 years (53%). It is also uncertain whether findings differed in part due to the racial/ethnic composition of participants between the two studies. The CHAMACOS Study comprises primarily Mexican-American children, whereas participants in the HOME Study consisted mainly of non-Hispanic white and black women. However, although the associations in the CHAMACOS Study did not reach statistical significance, point estimates for maternal PBDEs and BMI *z*-score at age 7 years did suggest a possible weak inverse association.

We did not observe effect measure modification by sex between PBDEs and measures of obesity. The only association that was significantly modified by child sex was between BDE-153 and height *z*-score. In the CHAMACOS Study, effect modification by child sex was consistent across *in utero* concentrations of BDE congeners and Σ4PBDEs (sum of BDEs 47, 99, 100, and 153) and BMI *z*-score, waist circumference, and obesity status (Erkin-Cakmak et al. 2015). The authors observed a positive association between maternal PBDEs and measures of obesity in males, but an inverse association in females. It is unclear whether child sex modifies the association between prenatal concentrations of PBDEs and child anthropometric measures, or what biological mechanisms could account for such differences.

Our findings do not support the role of maternal exposure to PBDEs as obesogens in early childhood. Still, the inverse association between maternal concentrations of BDE-153 and various measures of childhood obesity may be attributable to confounding by maternal adiposity. Similar to other lipophilic compounds, such as organochlorines, serum concentrations of PBDEs may be influenced by body weight (Chevrier 2013). Previous studies have reported significantly higher plasma and tissue concentrations of organochlorines following weight loss (Chevrier et al. 2000; Imbeault et al. 2001; Walford et al. 1999). It is posited that individuals who are heavier would have more adipose tissue for organochlorines and PBDEs to partition into, which would result in lower concentrations as a “dilution”of serum concentrations may occur (Glynn et al. 2003). The decrease in BMI *z*-score, waist circumference, and body fat percentage in children with higher prenatal PBDE concentrations could be attributable to this. Thus, an inverse association can be a result of higher PBDE concentrations in lean mothers. Although all BDE congeners and ΣPBDEs were inversely associated with BMI *z*-scores at 2–8 years and waist circumference at 4–8 years, only BDE-153 was statistically significant, perhaps due to BDE-153’s biochemical properties. Compared with the other BDE congeners, BDE-153 is more bioaccumulative, is the most difficult to metabolize and excrete, and has the highest fat deposition (Staskal et al. 2006). However, we did adjust by measures of maternal anthropometry and observed an inverse association between prenatal PBDEs and child anthropometric measures. This inverse association was present even without adjustment for maternal anthropometric measures, suggesting that PBDEs may not be obesogens.

Although some animal models have reported weight gain with PBDE exposure (Bondy et al. 2013; Dufault et al. 2005; Fernie et al. 2006; Gee and Moser 2008; Suvorov et al. 2009), others have indicated null or inverse associations (Daubié et al. 2011; Ta et al. 2011; Talsness et al. 2008). One possibility is that the direction of the association between PBDEs and growth measures differs between species. A reduction in thyroxine (T_4_) has been observed in rodents pre- and postnatally exposed to PBDEs across several studies (Kim et al. 2009; Kuriyama et al. 2007; Zhou et al. 2002), whereas some epidemiologic studies have reported an increase in T_4_ (Stapleton et al. 2011; Vuong et al. 2015). The precise biological mechanism involved in the observed inverse association between PBDEs and measures of childhood obesity is unclear. However, PBDEs’ mechanism of action may be similar to that of PCBs due to their similarities structurally and shared toxicological effects. PCBs have been observed to severely reduce the body weight of male rats in the neonatal period (Ahmed 2013). Male rats dosed with PCBs had decreased serum concentrations of growth hormone (GH) and IGF-1, suggesting a disruption of the GH/IGF-1 axis, which may delay growth. Further, decrements in body weight and fat mass may be attributed to the significantly higher concentrations of leptin, adiponectin, and tumor necrosis factor α (TNFα) that may have accelerated energy expenditure and lipid oxidation.

The strengths of the present study include its prospective design and long follow-up period. In addition to the multiple measures of weight, height, BMI, and waist circumference, we also measured body fat percentage at age 8 years. We accounted for a number of potential confounders, including sociodemographic factors, maternal depression, and maternal prepregnancy BMI. Concentrations of BDEs 28, 47, 99, and 100 were comparable between HOME Study participants and pregnant women in NHANES (National Health and Nutrition Examination Survey) 2003–2004, although concentrations of BDE-153 were somewhat lower (5.3 vs. 9.9 ng/g lipid) (see Table S1) (Woodruff et al. 2011). We also focused on PBDE exposures *in utero*, an important period of vulnerability, because developmental toxicants may perturb the central endocrine regulatory systems, which play a crucial role in long-term metabolic and body weight programming. In addition, confounding by other environmental toxicants purported as possible obesogens was also explored.

The findings of our study are subject to a number of limitations. Children in the HOME Study may have a lower BMI score than the national average. Approximately 13% and 8% of HOME Study children 2–8 years of age were overweight and obese, respectively. The prevalence of being overweight or obese among children in NHANES 1999–2012 is higher, with 23.8% and 11.1% of children 2–5 years, and 33.1% and 17.5% of those 6–11 years of age (Skinner and Skelton 2014). Further, HOME Study participants had a relatively high annual household income (~ $60,000) compared with the national median of ~ $48,500 in 2006 (Webster and Bishaw 2007). Thus, study findings may not be entirely generalizable to the U.S. population. Residual confounding may also be a concern because we did not have information on dietary intake and physical activity of the children. However, fresh fruit and vegetable intake during pregnancy was used as a proxy for dietary consumption of children as parental dietary patterns would influence caloric and nutrient intake of their children. Last, we did not examine postnatal exposures to PBDEs or PFASs, which may also influence measures of child anthropometry.

## Conclusion

Maternal serum concentrations of PBDEs during early pregnancy may alter measures of child anthropometry. In particular, BDE-153 was inversely associated with BMI *z*-score, waist circumference, and body fat percentage. Lower waist circumference was also observed in children with higher prenatal exposures to BDE-100 and ΣPBDEs. Our findings do not support the hypothesis that prenatal PBDE exposure is obesogenic in young children. In contrast, we found an inverse association between maternal PBDE concentrations in early pregnancy with child BMI *z*-score, waist circumference, and body fat percentage up to age 8 years. Further research is needed to replicate these findings while taking into account other environmental contaminants that could play a role in altering measures of child anthropometry.

## Supplemental Material

(528 KB) PDF
