# Supplementary material for: Prenatal Polybrominated Diphenyl Ether Exposure and Body Mass Index in Children Up To 8 Years of Age
Source: Environ Health Perspect. 2016 Jun 10;124(12):1891–7. doi: 10.1289/EHP139 (PMC5132628; doi:10.1289/EHP139)
Supplement: (528 KB) PDF [file EHP139.s001.acco.pdf]

Environ Health Perspect
DOI: 10.1289/EHP139

Note to readers with disabilities: *EHP* strives to ensure that all journal content is accessible to all readers. However, some figures and Supplemental Material published in *EHP* articles may not conform to 508 standards due to the complexity of the information being presented. If you need assistance accessing journal content, please contact ehp508@niehs.nih.gov. Our staff will work with you to assess and meet your accessibility needs within 3 working days.

Supplemental Material

Prenatal Polybrominated Diphenyl Ether Exposure and Body Mass Index in Children Up To 8 Years of Age

Ann M. Vuong, Joseph M. Braun, Andreas Sjödin, Glenys M. Webster, Kimberly Yolton, Bruce P. Lanphear, and Aimin Chen

Table of Contents

Table S1. Maternal concentrations of polybrominated diphenyl ether congeners (ng/g lipid), HOME Study

Table S2. Spearman rank correlation matrix for polybrominated diphenyl ethers (ng/g lipid), HOME Study

Table S3. Anthropometric measures in children at ages 1, 2, 3, 4, 5, and 8 years, HOME Study

Table S4. Comparing maternal and child characteristics of participants included and excluded in the examination of prenatal polybrominated diphenyl ethers and child anthropometric measures, HOME Study

Table S5. Odds ratios and 95% confidence intervals of being overweight or obese at 2, 3, 4, 5, and 8 years of age by 10-fold increases in maternal serum concentrations of polybrominated diphenyl ethers (ng/g lipid), HOME Study

Table S6. Odds ratios and 95% confidence intervals of having a BMI z-score ≤15th percentile at 2, 3, 4, 5, and 8 years of age by 10-fold increases in maternal serum concentrations of polybrominated diphenyl ethers (ng/g lipid), HOME Study

Table S7. Estimated differences and 95% confidence intervals in child anthropometric measures with 10-fold increases in maternal serum concentrations of polybrominated diphenyl ethers (ng/g lipid) by child sex, HOME Study

Table S8. Estimated differences and 95% confidence intervals in child anthropometric measures by 10-fold increases in maternal serum concentrations of polybrominated diphenyl ethers (ng/g lipid) with additional adjustment for environmental contaminants, HOME Study

Figure S1. Estimated differences and 95% CIs from multiple linear mixed models for associations between polybrominated diphenyl ether (ng/g lipid) tertiles and weight or height z-scores in children. All models adjusted for maternal age, race, education, income, maternal smoking status, maternal depression, and maternal fruit and vegetable intake during pregnancy. Weight z-score models were additionally adjusted for pre-pregnancy weight. Height z-score models were additionally adjusted for maternal height.

Table S1. Maternal concentrations of polybrominated diphenyl ether congeners[a] (ng/g lipid), HOME Study

PBDEs	*n*	Percent detection	Min	Percentile 25th	50th	75th	95th	Max	GM (GSD)	NHANES[d] GM (GSD)
∑PBDEs[b,c]	287	100.0	4.5	20.7	35.8	72.5	213.7	2046.9	39.1 (2.6)	-[e]
BDE-17	287	3.1	0.1	0.3	0.3	0.4	1.0	4.0	0.4 (1.6)	-
BDE-28[c]	287	80.1	0.2	0.6	1.0	1.8	4.6	31.4	1.1 (2.3)	-
BDE-47[c]	318	100.0	1.5	10.9	19.0	34.2	103	1290	20.3 (2.6)	23.9 (2.2)
BDE-66	287	1.7	0.1	0.3	0.3	0.4	1.1	2.6	0.3 (1.6)	-
BDE-85	287	47.4	0.2	0.3	0.5	1.0	3.5	38.7	0.6 (2.3)	-
BDE-99[c]	306	99.0	0.6	2.4	4.4	7.9	32.7	465	4.8 (2.8)	5.5 (0.8)
BDE-100[c]	287	97.9	0.4	2.1	3.5	8.1	25.4	172	4.0 (2.8)	6.1 (0.9)
BDE-153[c]	287	98.6	0.5	2.6	4.5	9.0	45.5	152	5.3 (2.9)	9.9 (3.0)
BDE-154	287	42.9	0.2	0.3	0.5	1.0	2.9	28.7	0.6 (2.3)	-
BDE-183	287	22.6	0.1	0.3	0.4	0.6	1.1	9.3	0.4 (1.8)	-

Abbreviations: GM, geometric mean; GSD, geometric standard deviation; Max, maximum; Min, minimum.

[a] Measured at 16±3 weeks of gestation

[b] Includes congeners with detection frequencies >80% (BDEs 28, 47, 99, 100, and 153)

[c] Included in statistical analyses

[d] Serum concentrations in pregnant women in NHANES 2003-2004, as reported by Woodruff et al

[e] Not available

Table S2. Spearman rank correlation matrix for polybrominated diphenyl ethers (ng/g lipid), HOME Study

PBDEs[a]	BDE-28	BDE-47	BDE-99	BDE-100	BDE-153
BDE-28					
BDE-47	0.86*				
BDE-99	0.75*	0.92*			
BDE-100	0.77*	0.90*	0.85*		
BDE-153	0.46*	0.50*	0.48*	0.73*	
∑PBDEs	0.82*	0.92*	0.88*	0.96*	0.73*

* $p<0.0001$.

[a] Total *n* for each of the pairwise correlations with BDE-28, -47, -99, -100, -153, and ∑PBDEs ranged from 287 to 306.

Table S3. Anthropometric measures in children at ages 1, 2, 3, 4, 5, and 8 years, HOME Study

	**Year**	***n***	**Mean±SD or %**
Weight z-scores	1	296	-0.29±1.17
	2	251	-0.07±1.19
	3	225	0.13±1.13
	4	174	0.21±1.13
	5	192	0.15±1.09
	8	216	0.39±1.10
Height z-scores	1	297	0.21±1.06
	2	249	0.07±1.04
	3	224	0.11±1.08
	4	173	0.28±1.14
	5	192	0.23±1.12
	8	216	0.24±1.14
BMI z-scores	2	246	0.02±1.03
	3	224	0.04±1.06
	4	173	0.15±1.13
	5	192	0.20±1.00
	8	216	0.35±0.98
Waist Circumference (cm)	4	171	51.9±4.4
	5	190	53.5±5.1
	8	211	60.7±8.2
Body Fat Percentage	8	210	20.8±6.1
Overweight or Obese[a]	2	41	16.7
	3	37	16.5
	4	31	17.9
	5	36	18.8
	8	53	24.5

Abbreviations: SD, standard deviation.

[a] BMI z-score ≥85th percentile

Table S4. Comparing maternal and child characteristics of participants included and excluded in the examination of prenatal polybrominated diphenyl ethers and child anthropometric measures, HOME Study

	Included		Excluded	
	**n**	**(%)[a]**	**n**	**(%)[a]**
Maternal age, years[b]				
<25	67	(21.1)	29	(40.9)
25-34	197	(62.0)	34	(47.9)
≥35	54	(17.0)	8	(11.3)
Race/ethnicity[b]				
Non-Hispanic White	211	(66.4)	26	(39.4)
Non-Hispanic Black and Others	107	(33.7)	40	(60.6)
Education[b]				
High school or less	73	(23.0)	27	(40.9)
Some college/2 yr degree	76	(23.9)	17	(25.8)
Bachelor's	102	(32.1)	8	(12.1)
Graduate or professional	67	(21.1)	14	(21.2)
Family Income[b]				
<$40,000	115	(36.2)	38	(57.6)
$40,000-$79,999	112	(35.2)	16	(24.2)
≥$80,000	91	(28.6)	12	(18.2)
Maternal Depression				
Minimal/mild	290	(91.8)	59	(90.8)
Moderate/severe	26	(8.2)	6	(9.2)
Maternal Smoking[b]				
None	274	(86.2)	47	(66.2)
ETS	21	(6.6)	14	(19.7)
Active	23	(7.2)	10	(14.1)
Prepregnancy BMI				
Underweight/Normal	166	(52.2)	39	(54.9)
Overweight	86	(27.0)	15	(21.1)
Obese	66	(20.8)	17	(23.9)
Marital status				
Married/living with partner	254	(79.9)	50	(75.8)
Not married, living alone	64	(20.1)	16	(24.2)
Fresh fruit and vegetable intake during pregnancy				
≥Daily	258	(81.1)	47	(71.2)
<Daily	60	(18.9)	19	(28.8)
Child Sex				
Male	146	(45.9)	35	(49.3)
Female	172	(54.1)	36	(50.7)

Abbreviations: GM, geometric mean; GSD, geometric standard deviation; SD, standard deviation.

[a] Frequencies may not add to the total number of participants because of missing values.

[b] $p < 0.05$

Table S5. Odds ratios and 95% confidence intervals of being overweight or obese[a] at 2, 3, 4, 5, and 8 years of age by 10-fold increases in maternal serum concentrations of polybrominated diphenyl ethers (ng/g lipid), HOME Study

PBDEs	*n*	Unadjusted OR (95% CI)	*n*	Adjusted[b] OR (95% CI)
BDE-28	263	1.59 (0.80, 3.15)	263	1.03 (0.50, 2.13)
BDE-47	289	1.66 (0.94, 2.95)	289	1.01 (0.54, 1.88)
BDE-99	279	1.43 (0.82, 2.50)	279	0.77 (0.41, 1.45)
BDE-100	263	1.37 (0.78, 2.42)	263	0.90 (0.49, 1.64)
BDE-153	263	0.63 (0.34, 1.13)	263	0.64 (0.35, 1.17)
∑PBDEs	263	1.34 (0.73, 2.49)	263	0.80 (0.41, 1.54)

PBDEs were $\log_{10}$-transformed

[a] BMI z-score ≥85th percentile

[b] Adjusted by maternal age, race, education, income, maternal smoking status, maternal depression, fresh fruit and vegetable intake during pregnancy, and pre-pregnancy BMI

Table S6. Odds ratios and 95% confidence intervals of having a BMI z-score ≤15th percentile at 2, 3, 4, 5, and 8 years of age by 10-fold increases in maternal serum concentrations of polybrominated diphenyl ethers (ng/g lipid), HOME Study

PBDEs	*n*	Unadjusted OR (95% CI)	*n*	Adjusted[a] OR (95% CI)
BDE-28	263	1.62 (0.76, 3.49)	263	1.81 (0.81, 4.04)
BDE-47	289	1.16 (0.59, 2.28)	289	1.35 (0.64, 2.83)
BDE-99	279	1.38 (0.74, 2.60)	279	1.92 (0.94, 3.94)
BDE-100	263	1.56 (0.82, 2.95)	263	1.88 (0.94, 3.73)
BDE-153	263	2.18 (1.22, 3.90)	263	2.18 (1.16, 4.08)
∑PBDEs	263	1.64 (0.82, 3.25)	263	2.02 (0.95, 4.31)

PBDEs were log_{10}-transformed

[a] Adjusted by maternal age, race, education, income, maternal smoking status, maternal depression, fresh fruit and vegetable intake during pregnancy, and pre-pregnancy BMI

Table S7. Estimated differences and 95% confidence intervals in child anthropometric measures[a] with 10-fold increases in maternal serum concentrations of polybrominated diphenyl ethers (ng/g lipid) by child sex, HOME Study

PBDEs	*p* for interaction	Adjusted[b]	
		Males β (95% CI)	Females β (95% CI)
Weight z-score[c]			
BDE-28	0.881	-0.25 (-0.74, 0.24)	-0.30 (-0.73, 0.13)
BDE-47	0.749	-0.13 (-0.54, 0.27)	-0.22 (-0.59, 0.15)
BDE-99	0.257	-0.10 (-0.47, 0.27)	-0.40 (-0.78, -0.02)
BDE-100	0.818	-0.24 (-0.64, 0.15)	-0.18 (-0.55, 0.18)
BDE-153	0.506	-0.33 (-0.69, 0.04)	-0.16 (-0.51, 0.19)
∑PBDEs	0.836	-0.24 (-0.67, 0.19)	-0.30 (-0.70, 0.09)
Height z-score[d]			
BDE-28	0.932	0.05 (-0.40, 0.50)	0.02 (-0.37, 0.42)
BDE-47	0.958	0.05 (-0.33, 0.42)	0.03 (-0.31, 0.38)
BDE-99	0.348	0.14 (-0.20, 0.49)	-0.09 (-0.43, 0.26)
BDE-100	0.591	-0.08 (-0.44, 0.29)	0.06 (-0.28, 0.40)
BDE-153	0.065*	-0.32 (-0.65, 0.01)	0.12 (-0.20, 0.44)
∑PBDEs	0.746	-0.06 (-0.45, 0.34)	0.03 (-0.33, 0.40)
BMI z-score[e]			
BDE-28	0.288	-0.40 (-0.87, 0.07)	-0.07 (-0.46, 0.32)
BDE-47	0.666	-0.15 (-0.54, 0.24)	-0.04 (-0.38, 0.30)
BDE-99	0.893	-0.20 (-0.56, 0.16)	-0.16 (-0.52, 0.19)
BDE-100	0.686	-0.26 (-0.64, 0.11)	-0.16 (-0.50, 0.18)
BDE-153	0.428	-0.26 (-0.60, 0.08)	-0.45 (-0.77, -0.13)
∑PBDEs	0.843	-0.29 (-0.69, 0.12)	-0.23 (-0.60, 0.14)
Waist Circumference[e,f]			
BDE-28	0.129	-2.70 (-5.37, -0.03)	-0.05 (-2.20, 2.09)
BDE-47	0.134	-2.17 (-4.34, -0.004)	-0.01 (-1.93, 1.92)
BDE-99	0.257	-2.23 (-4.28, -0.19)	-0.65 (-2.62, 1.33)
BDE-100	0.203	-2.50 (-4.59, -0.41)	-0.70 (-2.58, 1.18)
BDE-153	0.804	-2.01 (-4.01, -0.0001)	-1.67 (-3.39, 0.04)
∑PBDEs	0.191	-2.68 (-4.95, -0.40)	-0.67 (-2.71, 1.37)
Body Fat Percentage[e,f]			
BDE-28	0.345	-1.58 (-5.72, 2.56)	0.84 (-2.11, 3.78)
BDE-47	0.477	-1.54 (-5.08, 2.00)	-0.01 (-2.61, 2.59)
BDE-99	0.545	-1.92 (-5.21, 1.37)	-0.69 (-3.37, 1.98)
BDE-100	0.530	-1.90 (-5.07, 1.26)	-0.64 (-3.19, 1.91)
BDE-153	0.595	-1.81 (-4.58, 0.97)	-2.77 (-5.16, -0.39)
∑PBDEs	0.625	-2.20 (-5.81, 1.42)	-1.10 (-3.87, 1.68)

PBDEs were $\log_{10}$-transformed

[a] At 1-8 years of age for weight and height z-scores, 2-8 years of age for BMI z-scores, 4-8 years of age for waist circumference, and 8 years of age for body fat percentage

[b] Adjusted by maternal age, race, education, income, maternal smoking status, maternal depression, and fresh fruit and vegetable intake during pregnancy.

[c] Additionally adjusted by pre-pregnancy weight; [d] maternal height; [e] pre-pregnancy BMI; [f] child sex and child age

Table 8. Estimated differences and 95% confidence intervals[a] in child anthropometric measures[b] by 10-fold increases in maternal serum concentrations of polybrominated diphenyl ethers (ng/g lipid) with additional adjustment for environmental contaminants,[c] HOME Study

PBDEs	Weight z-scores[d] β (95% CI)	Height z-scores[e] β (95% CI)	BMI z-scores[f] β (95% CI)	Waist Circumference[f,g] β (95% CI)	Body Fat Percentage[f,g] β (95% CI)
PFOA[h]					
BDE-28	-0.29 (-0.62, 0.04)	-0.04 (-0.35, 0.26)	-0.21 (-0.52, 0.10)	-1.52 (-3.26, 0.21)	-0.42 (-2.93, 2.09)
BDE-47	-0.18 (-0.46, 0.10)	0.004 (-0.26, 0.27)	-0.09 (-0.36, 0.18)	-1.17 (-2.68, 0.35)	-0.90 (-3.12, 1.31)
BDE-99	-0.26 (-0.53, 0.02)	-0.004 (-0.26, 0.25)	-0.19 (-0.45, 0.08)	-1.52 (-3.01, -0.03)	-1.33 (-3.54, 0.88)
BDE-100	-0.22 (-0.49, 0.06)	-0.06 (-0.31, 0.20)	-0.21 (-0.47, 0.05)	-1.69 (-3.14, -0.24)	-1.44 (-3.51, 0.62)
BDE-153	-0.24 (-0.50, 0.02)	-0.14 (-0.38, 0.10)	-0.37 (-0.61, -0.13)	-1.99 (-3.33, -0.64)	-4.86 (-4.74, -0.97)
∑PBDEs	-0.28 (-0.58, 0.02)	-0.06 (-0.34, 0.22)	-0.27 (-0.55, 0.01)	-1.79 (-3.36, -0.22)	-1.83 (-4.13, 0.46)
PFOS[i]					
BDE-28	-0.31 (-0.65, 0.02)	-0.07 (-0.38, 0.23)	-0.22 (-0.53, 0.09)	-1.37 (-3.11, 0.37)	-0.37 (-2.91, 2.17)
BDE-47	-0.19 (-0.48, 0.10)	-0.02 (-0.29, 0.25)	-0.09 (-0.36, 0.18)	-1.06 (-2.58, 0.46)	-0.96 (-3.19, 1.28)
BDE-99	-0.26 (-0.54, 0.02)	-0.02 (-0.28, 0.23)	-0.18 (-0.45, 0.08)	-1.46 (-2.94, 0.03)	-1.37 (-3.60, 0.85)
BDE-100	-0.23 (-0.51, 0.05)	-0.09 (-0.34, 0.17)	-0.21 (-0.47, 0.04)	-1.56 (-3.01, -0.11)	-1.44 (-3.54, 0.65)
BDE-153	-0.26 (-0.52, 0.003)	-0.16 (-0.40, 0.08)	-0.38 (-0.62, -0.14)	-1.85 (-3.18, -0.52)	-2.77 (-4.67, -0.87)
∑PBDEs	-0.30 (-0.60, 0.005)	-0.09 (-0.37, 0.19)	-0.27 (-0.55, 0.01)	-1.66 (-3.23, -0.09)	-1.84 (-4.17, 0.48)
∑PCBs[j]					
BDE-28	-0.27 (-0.59, 0.05)	0.06 (-0.24, 0.35)	-0.17 (-0.48, 0.13)	-0.96 (-2.65, 0.74)	0.25 (-2.20, 2.70)
BDE-47	-0.17 (-0.45, 0.12)	0.09 (-0.17, 0.35)	-0.10 (-0.37, 0.18)	-0.98 (-2.54, 0.57)	-0.32 (-2.64, 2.01)
BDE-99	-0.22 (-0.50, 0.06)	0.08 (-0.18, 0.33)	-0.17 (-0.44, 0.09)	-1.38 (-2.90, 0.14)	-1.03 (-3.32, 1.26)
BDE-100	-0.20 (-0.46, 0.07)	0.02 (-0.23, 0.26)	-0.19 (-0.45, 0.06)	-1.42 (-2.85, 0.01)	-1.00 (-3.06, 1.05)
BDE-153	-0.23 (-0.48, 0.02)	-0.10 (-0.33, 0.13)	-0.35 (-0.59, -0.12)	-1.76 (-3.07, -0.45)	-2.30 (-4.15, -0.45)
∑PBDEs	-0.25 (-0.54, 0.04)	0.03 (-0.24, 0.29)	-0.24 (-0.52, 0.04)	-1.46 (-3.01, 0.10)	-1.32 (-3.62, 0.97)
DDE[k]					
BDE-28	-0.37 (-0.69, -0.04)	-0.02 (-0.31, 0.28)	-0.24 (-0.55, 0.07)	-1.37 (-3.07, 0.33)	-0.23 (-2.72, 2.25)
BDE-47	-0.22 (-0.49, 0.06)	0.02 (-0.24, 0.28)	-0.10 (-0.36, 0.16)	-1.11 (-2.60, 0.37)	-0.77 (-2.97, 1.43)
BDE-99	-0.26 (-0.53, 0.01)	0.03 (-0.22, 0.28)	-0.19 (-0.45, 0.07)	-1.50 (-2.98, -0.03)	-1.29 (-3.49, 0.92)
BDE-100	-0.25 (-0.52, 0.02)	-0.03 (-0.28, 0.22)	-0.22 (-0.48, 0.04)	-1.67 (-3.10, -0.24)	-1.36 (-3.43, 0.71)
BDE-153	-0.24 (-0.49, 0.01)	-0.10 (-0.33, 0.13)	-0.36 (-0.60, -0.13)	-1.85 (-3.15, -0.54)	-2.45 (-4.29, -0.60)
∑PBDEs	-0.31 (-0.60, -0.02)	-0.02 (-0.29, 0.24)	-0.27 (-0.55, 0.01)	-1.72 (-3.27, -0.18)	-1.73 (-4.02, 0.56)

DEHP[l]					
BDE-28	-0.30 (-0.63, 0.03)	-0.02 (-0.33, 0.28)	-0.23 (-0.54, 0.08)	-1.54 (-3.22, 0.15)	0.09 (-2.41, 2.60)
BDE-47	-0.19 (-0.47, 0.09)	-0.01 (-0.27, 0.26)	-0.10 (-0.36, 0.17)	-1.19 (-2.66, 0.28)	-0.59 (-2.81, 1.63)
BDE-99	-0.26 (-0.53, 0.02)	-0.001 (-0.25, 0.25)	-0.19 (-0.45, 0.08)	-1.54 (-3.00, -0.08)	-1.12 (-3.34, 1.11)
BDE-100	-0.23 (-0.50, 0.05)	-0.04 (-0.29, 0.21)	-0.23 (-0.48, 0.03)	-1.69 (-3.09, -0.28)	-1.24 (-3.31, 0.82)
BDE-153	-0.27 (-0.53, -0.01)	-0.12 (-0.35, 0.11)	-0.40 (-0.63, -0.16)	-2.02 (-3.31, -0.73)	-2.57 (-4.43, -0.71)
∑PBDEs	-0.30 (-0.59, 0.001)	-0.05 (-0.32, 0.22)	-0.28 (-0.56, -0.002)	-1.80 (-3.33, -0.27)	-1.60 (-3.91, 0.70)
BPA[m]					
BDE-28	-0.29 (-0.62, 0.03)	-0.03 (-0.33, 0.28)	-0.21 (-0.52, 0.10)	-1.42 (-3.12, 0.28)	0.21 (-2.29, 2.70)
BDE-47	-0.19 (-0.47, 0.10)	-0.01 (-0.27, 0.26)	-0.08 (-0.35, 0.19)	-1.11 (-2.61, 0.38)	-0.32 (-2.54, 1.91)
BDE-99	-0.25 (-0.53, 0.02)	-0.005 (-0.26, 0.25)	-0.17 (-0.43, 0.10)	-1.45 (-2.93, 0.03)	-0.81 (-3.06, 1.43)
BDE-100	-0.23 (-0.50, 0.05)	-0.05 (-0.31, 0.20)	-0.21 (-0.47, 0.05)	-1.69 (-3.13, -0.26)	-0.94 (-3.02, 1.14)
BDE-153	-0.27 (-0.53, -0.01)	-0.14 (-0.37, 0.10)	-0.38 (-0.62, -0.14)	-2.07 (-3.39, -0.75)	-2.33 (-4.21, -0.45)
∑PBDEs	-0.30 (-0.59, 0.003)	-0.06 (-0.34, 0.21)	-0.26 (-0.54, 0.02)	-1.78 (-3.34, -0.22)	-1.30 (-3.62, 1.02)

PBDEs were $\log_{10}$-transformed

[a] Adjusted by maternal age, race, education, income, maternal smoking status, maternal depression, fresh fruit and vegetable intake during pregnancy

[b] At 1-8 years of age for weight and height z-scores, 2-8 years of age for BMI z-scores, 4-8 years of age for waist circumference, and 8 years of age for body fat percentage

[c] Maternal concentrations at 16±3 weeks of gestation

[d] Additionally adjusted by pre-pregnancy weight; [e] maternal height; [f] pre-pregnancy BMI; [g] child sex and child age

[h] Models of child anthropometric measures additionally adjusted by maternal concentrations of PFOA; [i] PFOS; [j] ∑PCBs; [k] DDE; [l] DEHP; and [m] BPA.

Figure S1. Estimated differences and 95% CIs from multiple linear mixed models for associations between polybrominated diphenyl ether (ng/g lipid) tertiles and weight or height z-scores in children. All models adjusted for maternal age, race, education, income, maternal smoking status, maternal depression, and maternal fruit and vegetable intake during pregnancy. Weight z-score models were additionally adjusted for pre-pregnancy weight. Height z-score models were additionally adjusted for maternal height.

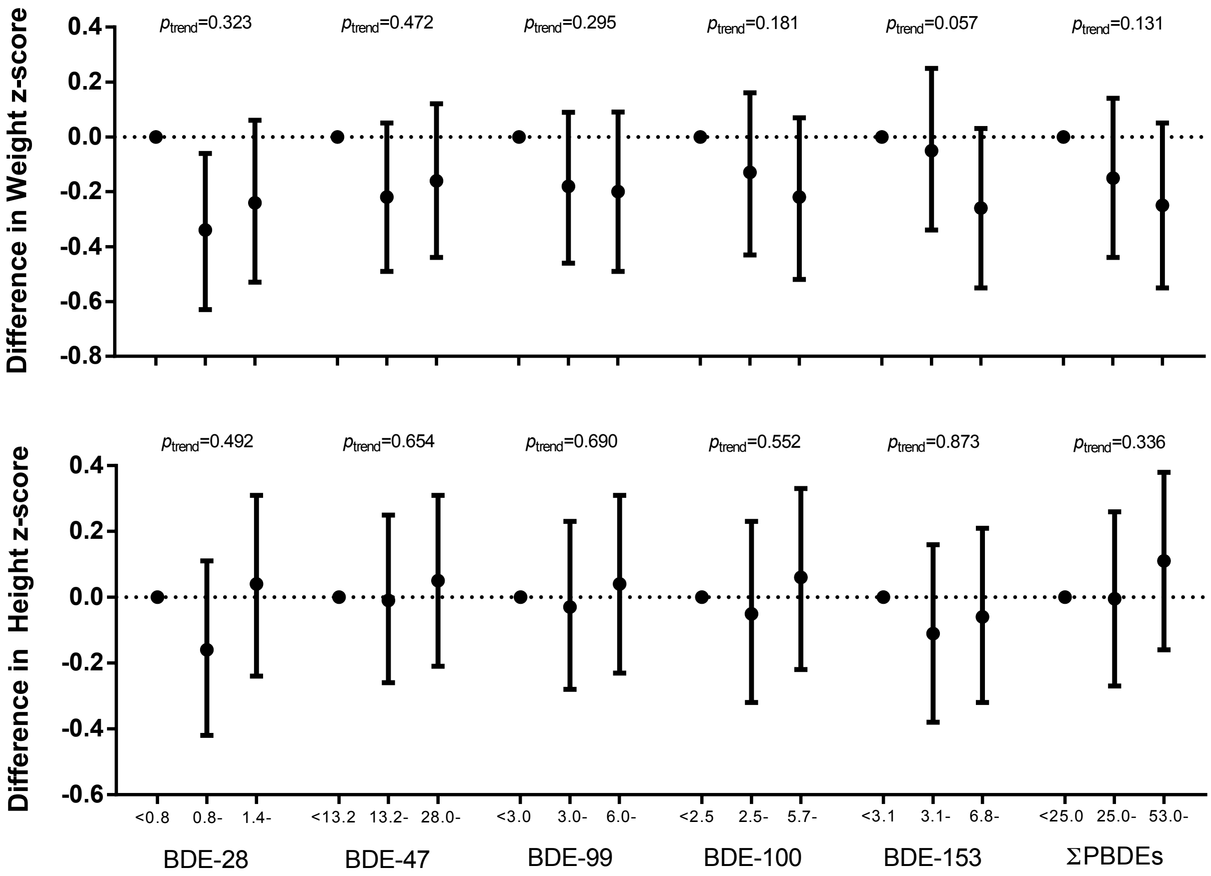

Reference

Woodruff TJ, Zota AR, Schwartz JM. 2011. Environmental chemicals in pregnant women in the United States: NHANES 2003-2004. Environ Health Perspect 119:878-885.
